# Preparation, Structural Characterization of Octenyl Succinic Anhydride-Modified Bamboo Shoot-Derived Cellulose Nano-Crystals

**DOI:** 10.3390/foods14223876

**Published:** 2025-11-13

**Authors:** Maokun Huang, Wen Chen, Zichen Zhang, Qi Wang, Yunlong Li, Yafeng Zheng

**Affiliations:** 1 School of Light Industry, Liming Vocational University, Quanzhou 362000, China; 20082726@lmu.edu.cn; 2 Fujian Higher Education Engineering Research Center for Applied Technology in Marine Biomass Resources Development, Quanzhou 362000, China; 3 College of Food Science, Fujian Agriculture and Forestry University, Fuzhou 350002, China; wenchen042@gmail.com (W.C.); 18395026998@163.com (Z.Z.); 4 Institute of Food Science and Technology, Fujian Academy of Agricultural Sciences, Fuzhou 350003, China; faaswq@163.com

**Keywords:** bamboo shoot by-products, cellulose nanocrystals (CNCs), octenyl succinic anhydride (OSA) modification, emulsion stabilizer

## Abstract

To tackle the poor emulsibility of hydrophilic cellulose nanocrystals (CNCs), this study prepared octenyl succinic anhydride (OSA)-modified bamboo shoot CNC (OSNC) via acid hydrolysis and esterification, using microcrystalline cellulose (MCC) as a control. The degree of substitution (DS), chemical structure, crystalline structure, morphological characteristics, zeta potential, wettability, and thermal stability of OSNC and OSA-modified MCC (OSA-MCC) were characterized using multiple techniques. Results showed that at the optimal cellulose-to-OSA ratio (1:0.225), OSNC had a higher DS (0.029 ± 0.01) than OSA-MCC (0.024 ± 0.02). FTIR confirmed successful OSA grafting; XRD showed a preserved cellulose I crystal form with slightly reduced crystallinity; OSNC had improved dispersion stability (zeta potential: −44.0 mV), balanced amphiphilicity (contact angle: 61.8°), and enhanced thermal stability. This work enables high-value utilization of bamboo shoot by-products and supports developing green food-grade cellulose-based nanomaterials for food emulsions.

## 1. Introduction

As the world’s largest producer and exporter of bamboo shoots, China plays a leading role in the global bamboo shoot market. Bamboo shoots are extensively acknowledged for their health-promoting properties, primarily attributed to their nutritional profile: high dietary fiber content, low fat content, rich protein supply, and a wealth of bioactive compounds [1,2]. Fresh bamboo shoots are susceptible to oxidative browning and lignification and have a short marketing cycle; thus, only approximately 40% of them are sold as fresh products, while most are processed into dried bamboo shoots, sour bamboo shoots and canned products. This processing process generates substantial by-products such as bamboo shoot bases and shells, which account for more than 65% of the total weight of raw bamboo shoots [2]. Currently, these by-products are mostly discarded except for a small amount used as animal feed and textile fibers, resulting in resource waste and environmental pollution. Notably, the insoluble cellulose content in bamboo shoot bases is about 48.3–69.78%, which can serve as a high-quality raw material for the preparation of nanocellulose [3].

Cellulose nanocrystals (CNC) are the smallest structural units prepared from cellulose, with a needle-like structure, a diameter of 1–50 nm and a length of 100–500 nm [4]. They have gained tremendous attention in food, packaging, and biomedical fields due to their renewability, biodegradability, high mechanical strength, and excellent biocompatibility [5,6]. Acid hydrolysis is the most widely used method for CNC preparation, as it efficiently removes the amorphous regions of cellulose while retaining the crystalline structure. However, pristine CNC exhibits strong hydrophilicity due to abundant surface hydroxyl groups, which limits its dispersion in hydrophobic media and adsorption at the oil-water interface—key properties for applications such as emulsion stabilization [7]. To address this limitation, surface modification of CNC is essential to introduce hydrophobic groups, thereby endowing it with amphiphilicity and expanding its practical applicability.

The surface chemical modification methods of CNCs are generally categorized into two main types: small-molecule-based chemical modification and surface grafting of macromolecules [8]. Among these strategies, small-molecule esterification is particularly valued for its simplicity, mild reaction conditions, and adaptability to functional customization. Octenyl succinic anhydride (OSA) esterification is a well-established and food-grade modification method for polysaccharides [9,10]. Under alkaline conditions, the free hydroxyl groups on cellulose react with OSA, introducing both hydrophilic carboxylate groups (-COO^−^) and hydrophobic long-chain alkenyl groups onto the molecular chain. This dual modification balances the hydrophilic-hydrophobic properties of cellulose, significantly improving its interfacial activity and emulsifying ability [11]. Previous studies have demonstrated the successful application of OSA modification to a range of solid particles—such as starch nanoparticles (corn, rice) [12,13], bulk cellulose (sweet potato residue) [7] and cotton CNCs [11]—a strategy that effectively enhances their potential for acting as emulsion stabilizers. However, research on OSA modification of bamboo shoot-derived CNC remains scarce. Given the unique structural characteristics of bamboo shoot cellulose, its OSA modification efficiency and the performance of the resulting OSA-modified bamboo shoot CNC (OSNC) may differ significantly from that of commercial cellulose (e.g., microcrystalline cellulose, MCC) or other plant-derived CNCs, warranting systematic investigation.

To promote the high-value utilization of bamboo shoot processing by-products and develop sustainable cellulose-based nanomaterials, this study first prepared CNCs from bamboo shoot bases via a two-step acid hydrolysis-ultrasound method, followed by OSA modification to obtain OSNC. Commercial MCC was used as a control to benchmark modification efficiency and performance. The key properties of OSNC and OSA-MCC (OSA-modified MCC) were comprehensively characterized, including degree of substitution (DS), chemical structure (Fourier transform infrared spectroscopy, FTIR), crystalline structure (X-ray diffraction, XRD), morphology (scanning electron microscopy, SEM), zeta potential, wettability (contact angle measurement), and thermal stability. The specific research objectives were: (1) to clarify how OSA modification alters the structural and physicochemical properties of bamboo shoot-derived CNCs; (2) to compare the modification efficiency (DS) and functional performance (e.g., amphiphilicity, thermal stability) of OSNC with OSA-MCC, quantifying differences driven by bamboo CNC’s intrinsic features; and (3) to lay a theoretical foundation for the application of OSNC as a green, food-grade Pickering emulsion stabilizer—filling the gap of bamboo-specific nanocellulose modification in existing literature. This work not only addresses the environmental issue of bamboo shoot by-product waste but also provides a new candidate material for sustainable food and cosmetic formulations, aligning with the global goals of circular agriculture and green manufacturing.

## 2. Materials and Methods

### 2.1. Materials

Fresh green bamboo shoots were purchased from local supermarkets in Fuzhou, Fujian Province, and their basal parts (bamboo shoot bases) were used as raw materials after removal. Octenyl succinic anhydride (OSA) was obtained from Sigma-Aldrich (St. Louis, MO, USA). Microcrystalline cellulose (MCC), also purchased from Sigma-Aldrich, was used as a control group. Anhydrous ethanol, sulfuric acid (H_2_SO_4_), hydrochloric acid (HCl), and sodium hydroxide (NaOH) were supplied by Xilong Scientific Co., Ltd. (Shantou, China). Hydrogen peroxide (H_2_O_2_, 30% *v*/*v*) was obtained from Sinopharm Chemical Reagent Co., Ltd. (Shanghai, China). All reagents used were of analytical grade unless otherwise specified.

### 2.2. Preparation of Bamboo Shoot CNC

Bamboo shoot-derived CNC was prepared via acid hydrolysis with modifications based on the method described by Lin et al. [5]. First, the bamboo shoot bases were cut into small pieces, dried in a blast oven at 45 °C for 48 h, and ground into powder. The powder was mixed with ethyl acetate at a ratio of 1:4 (g/mL) for 1 h to degrease, followed by washing with deionized water 5 times. The degreased sample was then mixed with deionized water at 1:30 (g/mL) and stirred at 80 °C for 4 h; after cooling, it was centrifuged at 4000 rpm for 10 min, and the residue was washed with anhydrous ethanol, dried, and sieved through an 80-mesh sieve to obtain crude insoluble dietary fiber (IDF).

For bleaching, the IDF was mixed with 5% NaOH (1:20, g/mL) and stirred at 80 °C for 4 h, then centrifuged (4000 rpm, 10 min) and washed 4 times. The residue was soaked in 8% H_2_O_2_ (1:20, g/mL) for 30 h, followed by centrifugation (4000 rpm, 5 min) and washing with deionized water 4 times to remove residual H_2_O_2_. Bamboo shoot-derived cellulose nanofibers (CNFs) were prepared via two-step acid hydrolysis combined with ultrasound: the first acid hydrolysis was performed with 5% HCl at 180 W ultrasound for 90 min, and the second with 10% HCl at 170 W ultrasound for 60 min (both steps at 45 °C, 1:30 g/mL solid–liquid ratio). The hydrolyzed residue was washed with deionized water to pH 7.0, freeze-dried, and ground to obtain CNFs.

Finally, 3 g of CNFs was mixed with 62 wt% H_2_SO_4_ (1:20, g/mL) and reacted at 39 °C for 45 min. Ten volumes of deionized water were added to terminate the reaction, and the mixture was centrifuged at 10,000 rpm for 5 min to remove excess H_2_SO_4_. The turbid supernatant was dialyzed against deionized water for 6–7 days to remove residual acid, yielding a bamboo shoot-derived CNC suspension, which was stored at 4 °C for subsequent use.

### 2.3. OSA Modification of Bamboo Shoot CNC

OSA-modified bamboo shoot CNC (OSNC) and OSA-modified MCC (OSA-MCC) were prepared with slight modifications based on a reported method [14]. First, a preliminary screening experiment was conducted to determine the optimal OSA dosage: 2.0 wt% CNC suspensions were separately prepared, magnetically stirred for 5 min, and sonicated at 160 W for 10 min to ensure uniform dispersion. OSA was then added to the CNC suspensions at different mass ratios of CNC to OSA (1:0.175, 1:0.200, 1:0.225 and 1:0.250), with the reaction temperature consistently maintained at 25 °C. For each group, the pH of the mixture was adjusted to 8.5 ± 0.1 using 0.5 mol/L NaOH and kept stable by continuous NaOH addition until no further pH decline was observed (total reaction duration: 7 h). After reaction, the mixtures were neutralized to pH 7.0 with 0.5 mol/L HCl, centrifuged at 10,000 rpm for 10 min, and the precipitates were washed 3 times with deionized water and 3 times with anhydrous ethanol to remove residual OSA. The resulting suspensions were dialyzed for 3 days to eliminate ethanol, freeze-dried, and ground into powders. Ultimately, the 1:0.225 (cellulose:OSA) mass ratio was determined to be the optimal dosage, and this ratio was therefore adopted for the preparation of both OSNC and OSA-MCC used in subsequent experiments.

### 2.4. Characterization of Modified and Unmodified Cellulose Particles

#### 2.4.1. Degree of Substitution (DS) Determination

The DS was measured via alkali hydrolysis-acid back titration [15]. A 20 mL aliquot of 0.1 wt% particle suspension was magnetically stirred for 30 min, and a defined volume of 0.5 mol/L NaOH was added; the mixture was stirred for 6 h to ensure complete hydrolysis. Phenolphthalein was added as an indicator, and the excess NaOH was titrated with 0.1 mol/L HCl until the solution turned colorless. Unmodified CNCs and MCC were used as blanks. The DS was calculated according to the following equation:

(1) DS = (162 × n_COOH_)/(m − 210 × n_COOH_)

where 162 g/mol is the molar mass of the anhydroglucose unit (AGU), 210 g/mol is the net molar mass increase in AGU per acyl substitution, m is the mass of the sample (g), and n_COOH_ is the molar amount of carboxyl groups (equal to the molar amount of consumed NaOH, mol).

#### 2.4.2. Fourier Transform Infrared Spectroscopy (FTIR) Analysis

Freeze-dried powder samples (10 mg) were mixed with KBr (1:100, *w*/*w*), ground, and pressed into pellets. FTIR spectra were recorded using a Nicolet iS50 FTIR spectrometer (Thermo Fisher Scientific, Waltham, MA, USA) in the range of 400–4000 cm^−1^ with a resolution of 4 cm^−1^ and 64 scans. To quantify the esterification extent, peak fitting was performed using OriginPro 2023, and ratios of ester carbonyl peak intensity at 1724 cm^−1^ to the cellulose skeletal vibration peak at 1431 cm^−1^ (A_1724_/A_1431_) was calculated.

#### 2.4.3. X-Ray Diffraction (XRD) Analysis

The crystal structure of the samples was analyzed using an Ultima IV X-ray diffractometer (Rigaku, Tokyo, Japan) with Cu Kα radiation (λ = 0.154 nm) at 30 kV and 30 mA. The scanning range was 5–70° (2θ) with a scanning speed of 2 °C/min and step size of 0.02°. The crystallinity index (*CrI*) was calculated via Segal method according to the following equation:

(2) *CrI* = [(*I*_22.3_ − *I_am_*)/*I*_22.3_] × 100%

where *I*_22.3_ is the maximum intensity at 2θ ≈ 22.3° (crystalline region), and *I_am_* is the intensity at 2θ ≈ 18° (amorphous region).

#### 2.4.4. Scanning Electron Microscopy (SEM) Observation

Sample suspensions were diluted to 0.001 wt% and sonicated for 10 min to disperse uniformly. A 10 μL aliquot of the diluted suspension was dropped onto a clean mica sheet, dried at room temperature, and sputter-coated with gold. The morphology of the particles was observed using a SU8010 field-emission SEM (Hitachi, Tokyo, Japan) at an acceleration voltage of 10 kV. The particle size was measured using Nano Measurer 1.2 software (*n* = 50).

#### 2.4.5. Zeta Potential Analysis

Sample suspensions were diluted to 0.02 wt% and sonicated for 10 min. The Zeta potential was measured using a Zetasizer Ultra (Malvern Panalytical, Malvern, UK) at 25 °C with a scattering angle of 90°. Each sample was measured in triplicate, and the results were expressed as mean ± standard deviation.

#### 2.4.6. Contact Angle Measurement

A JY-82B Kruss DSA contact angle analyzer (Dataphysics, Filderstadt, Germany) was used to measure the water contact angle of the samples at 25 °C. Freeze-dried powder was pressed into discs (13 mm diameter, 2 mm thickness). A 5 μL droplet of deionized water was deposited onto the disc surface using a microsyringe, and the contact angle was analyzed using the instrument software. Each sample was measured in triplicate.

#### 2.4.7. Thermogravimetric Analysis (TGA)

Thermal stability was evaluated using a TG209F3 TGA analyzer (Netzsch, Selb, Germany). Approximately 5 mg of freeze-dried sample was heated from 30 °C to 790 °C at a rate of 10 °C/min under a nitrogen atmosphere (flow rate: 20 mL/min), and the mass loss was recorded.

### 2.5. Statistical Analysis

All experiments were performed in triplicate, and the results were expressed as mean ± standard deviation. Statistical analysis was conducted using SPSS 19.0 software (IBM, Armonk, NY, USA), and significant differences (*p* < 0.05) were determined using Duncan’s multiple range test.

## 3. Results and Discussion

### 3.1. Degree of Substitution (DS)

The degree of substitution (DS) is a core indicator for quantifying octenyl succinic anhydride (OSA) grafting efficiency onto cellulose, directly governing the hydrophilic-hydrophobic balance of modified particles and their interfacial/emulsifying performance. Table 1 presents DS values of OSNC under four CNC-to-OSA ratios to optimize the modification process. As shown in Table 1, OSNC’s DS increased significantly then plateaued with higher OSA dosage. When the CNC-to-OSA ratio decreased from 1:0.175 to 1:0.225, DS rose from 0.019 ± 0.02 to 0.029 ± 0.01 (*p* < 0.05), as more OSA groups reacted with CNC surface hydroxyls. However, increasing the ratio to 1:0.250 only slightly raised DS to 0.030 ± 0.02 (*p* > 0.05), indicating CNC surface reactive sites neared saturation, and excess OSA adds no efficiency but increases cost and residual risk. Notably, at 1:0.225, OSNC’s DS (0.029 ± 0.01) was significantly higher than OSA-MCC’s (0.024 ± 0.02, *p* < 0.05), and also higher than the DS range of OSA-modified sweet potato residue cellulose (OSA-SPRC: 0.0017 ± 0.0003 to 0.0063 ± 0.0005) [7]. This is due to bamboo shoot-derived CNC’s structural advantages: more exposed hydroxyls (from acid hydrolysis removing amorphous regions) and larger specific surface area (nanoscale morphology), enhancing OSA accessibility and reactivity. All modified products’ DS (0.019–0.030) falls within the 0.02–0.05 range for food-grade OSA-modified cellulose [7], ensuring food safety (avoiding off-flavors or reduced biocompatibility from excessive DS). Combined with subsequent contact angle data, this DS also endows OSNC with balanced amphiphilicity, supporting its use as a Pickering emulsion stabilizer. Considering DS, efficiency, cost, and application potential, the 1:0.225 CNC-to-OSA ratio was selected as optimal. OSNC and OSA-MCC prepared under this condition were used for further characterization of wettability, thermal stability, and emulsifying performance.

### 3.2. Fourier Transform Infrared Spectroscopy (FTIR) Analysis

**Figure 1:**
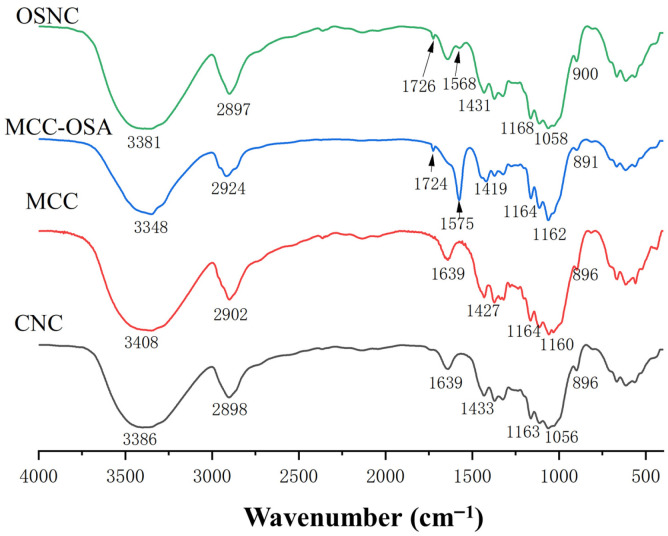
Comparative FTIR spectra of bamboo shoot-derived CNC, MCC, OSA-modified MCC (OSA-MCC), and OSA-modified CNC (OSNC).

FTIR was employed to verify the successful introduction of OSA groups onto the cellulose surface and changes in functional groups [16]. Figure 1 shows the FTIR spectra of unmodified bamboo shoot CNC, MCC, and their OSA-modified derivatives (OSNC and OSA-MCC). For unmodified cellulose samples, the broad and strong absorption peak around 3380 cm^−1^ corresponds to the stretching vibration of -OH groups, a typical characteristic of cellulose [17]. After OSA modification, the intensity of this -OH peak in OSNC and OSA-MCC significantly decreased. This reduction is due to the esterification reaction between hydroxyl groups on the cellulose surface and OSA, which consumes free -OH groups to form ester bonds, confirming the occurrence of chemical modification [18]. Two new characteristic absorption peaks appeared in the spectra of OSNC and OSA-MCC: a peak at 1724 cm^−1^ attributed to the stretching vibration of ester carbonyl groups (C=O) and a peak at 1568 cm^−1^ corresponding to the asymmetric stretching vibration of carboxylate anions (RCOO^−^) [19], directly confirming successful OSA grafting. To quantify the esterification extent, the ratios of the carbonyl peak (1724 cm^−1^) to the cellulose skeletal vibration peak (1431 cm^−1^, C-H bending), denoted as A_1724_/A_1431_, were calculated: OSNC exhibited a ratio of 0.87 ± 0.03, significantly higher than OSA-MCC (0.52 ± 0.02, *p* < 0.05).

Notably, no obvious absorption peaks of cyclic anhydrides (around 1775 cm^−1^ and 1858 cm^−1^) were observed in the spectra of OSNC and OSA-MCC, indicating the absence of residual OSA in the modified products. This is crucial for ensuring the safety of subsequent applications in food systems, as residual OSA may cause adverse effects such as acidity or off-flavors. Other characteristic peaks of cellulose remained unchanged after modification: peaks at 2905 cm^−1^ and 1431 cm^−1^ correspond to the stretching and bending vibrations of -CH_2_ groups, respectively; the peak at 1647 cm^−1^ is due to the stretching vibration of alkenyl groups in cellulose; and the peaks at 1168 cm^−1^, 1060 cm^−1^, and 893 cm^−1^ are related to the stretching vibration of C-O-C, the asymmetric “bridge” stretching vibration of C-O, and the skeletal vibration of the anhydroglucose ring (β-glycosidic bond), respectively [20]. These results confirm that OSA esterification does not destroy the basic molecular structure of cellulose, ensuring the retention of its inherent advantages such as biocompatibility and mechanical strength.

The mechanism of OSA grafting onto bamboo shoot-derived CNC follows a nucleophilic acyl substitution pathway [11]. Under alkaline conditions (pH 8.5), surface hydroxyls of CNC deprotonate to alkoxide ions, attacking the electrophilic carbonyl of OSA’s anhydride ring. This cleaves the ring, forming ester bonds and carboxylate groups. Kinetically, DS increases with OSA dosage until hydroxyl saturation (as shown in Table 1). Thermodynamically, ester bond formation (ΔG < 0) drives the reaction. Structurally, OSA preferentially reacts with C6 primary hydroxyl groups of cellulose due to their lower steric hindrance—an observation supported by both FTIR (reduced -OH peak intensity) and following XRD (preserved cellulose Type I crystal form) results.

### 3.3. X-Ray Diffraction (XRD) Analysis

**Figure 2:**
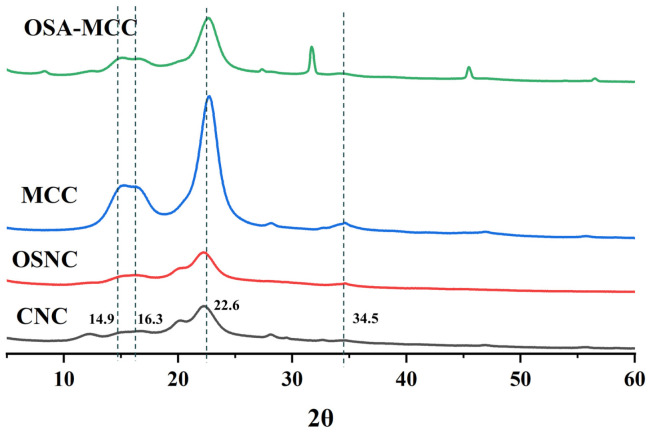
XRD patterns of bamboo shoot-derived CNC, MCC, OSA-modified MCC (OSA-MCC), and OSA-modified CNC (OSNC).

XRD analysis was conducted to investigate the effect of OSA modification on the crystalline structure of cellulose particles. Figure 2 shows the XRD patterns of unmodified and modified cellulose samples. All cellulose samples exhibit the characteristic diffraction peaks of cellulose Type I, with corresponding 2θ values of 14.9°, 16.3°, 22.6°, and 34.5°, indicating that OSA modification does not alter the crystal form of cellulose [21]. Notably, the retention of the cellulose Type I crystal form after OSA modification is of great significance: it ensures the maintenance of mechanical stability and biodegradability of the modified particles, which are essential properties for their application as Pickering emulsion stabilizers. The crystallinity index (*CrI*) of the samples was calculated using the Segal method. After OSA modification, the *CrI* of OSA-MCC decreased from 87.58 ± 0.01% (unmodified MCC) to 81.17 ± 0.03% (*p* < 0.05), while the *CrI* of OSNC decreased from 82.76 ± 0.04% (unmodified bamboo shoot CNC) to 75.12 ± 0.01% (*p* < 0.05). This reduction in crystallinity can be explained by the reaction mechanism of OSA esterification: OSA molecules primarily react with hydroxyl groups in the amorphous regions and on the surface of crystalline regions of cellulose. The introduction of OSA long-chain alkenyl groups disrupts the ordered arrangement of cellulose molecular chains in the amorphous regions, reducing the degree of crystallization [22]. However, the slight decrease in crystallinity does not compromise the structural integrity of the particles, as evidenced by the retention of the main diffraction peaks. Instead, the reduced crystallinity may enhance the flexibility of OSNC particles, facilitating their adsorption and arrangement at the oil-water interface.

### 3.4. Scanning Electron Microscopy (SEM) Analysis

**Figure 3:**
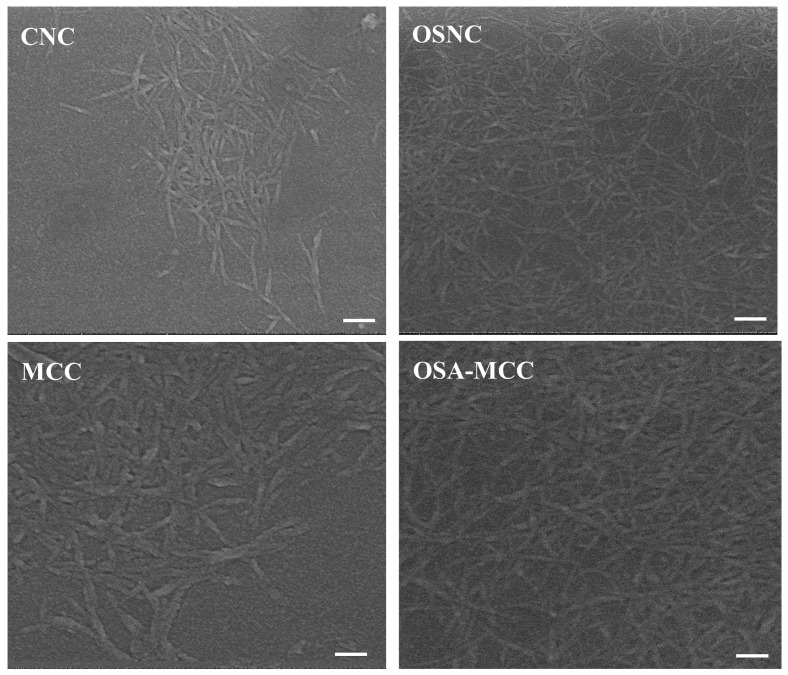
SEM images of bamboo shoot-derived CNC, OSA-modified CNC (OSNC), MCC, and OSA-modified MCC (OSA-MCC); scale of CNC and OSNC is 100 nm, and scale of MCC and OSA-MCC is 100 μm.

SEM was used to observe the morphological changes in cellulose particles before and after modification, as particle morphology directly affects their interfacial adsorption behavior. Figure 3 shows the SEM images of unmodified bamboo shoot CNC, MCC, and their OSA-modified derivatives. Bamboo shoot CNC exhibits a needle-like nanostructure with a highly uniform morphology, with an average diameter of 28.47 ± 7.05 nm and a length of 282.86 ± 20.13 nm, presenting a dispersed or loosely aggregated state due to intermolecular hydrogen bonding. MCC appears as irregularly shaped, rod-like particles with a relatively smooth surface, with an average diameter of 16.05 ± 1.16 μm and a length of 152.43 ± 19.43 μm.

After OSA modification, the average diameters of OSNC and OSA-MCC increased, mainly due to the mild aggregation of particles caused by the introduction of hydrophobic groups. The hydrophobic alkenyl chains introduced by OSA tend to form hydrophobic microenvironments between adjacent particles, leading to weak intermolecular aggregation [23]. This aggregation is not a change in the particle itself but a result of surface interaction, which is beneficial for improving the adsorption capacity of particles at the oil-water interface—aggregated particles can form a more compact interface film, enhancing the mechanical barrier effect. The needle-like morphology of OSNC is particularly advantageous for its application as a Pickering emulsion stabilizer. Compared with spherical particles, needle-like particles can interlock with each other at the oil-water interface, forming a more rigid and dense interface film. Additionally, no significant changes in particle surface smoothness were observed after modification, indicating that OSA modification does not cause severe damage to the particle surface, ensuring the retention of its structural integrity.

### 3.5. Zeta Potential Analysis

**Figure 4:**
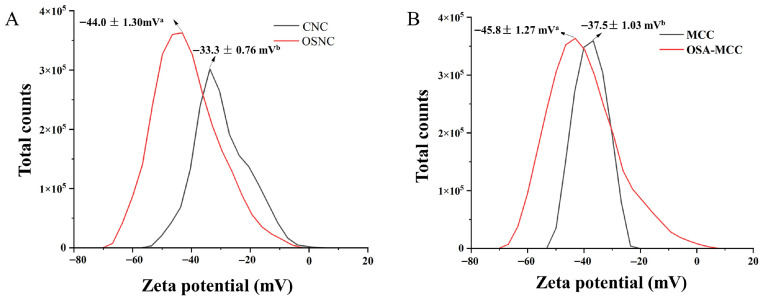
Zeta potentials of bamboo shoot-derived CNC and OSA-modified CNC (OSNC, (**A**)), as well as MCC and OSA-modified MCC (OSA-MCC, (**B**)). Different lowercase letters (a, b) indicate significant differences (*p* < 0.05) between unmodified and OSA-modified samples.

The zeta potential of the particles reflects their surface charge density, which directly affects the electrostatic repulsion between particles. Figure 4 shows the zeta potential of unmodified and modified cellulose samples. The zeta potential of unmodified bamboo shoot CNC is −33.3 ± 0.76 mV, and that of MCC is −37.5 ± 1.03 mV. These negative potentials are due to the sulfate groups introduced on the particle surface during acid hydrolysis [24]. After OSA modification, the zeta potential of OSA-MCC significantly increases to −45.8 ± 1.27 mV, and that of OSNC increases to −44.0 ± 1.30 mV (*p* < 0.05), which is comparable to that of OSA-modified cotton CNC (−44.4 mV to −45.6 mV) [11]. The significant increase in the absolute value of zeta potential is attributed to the introduction of carboxyl groups (-COO^−^) by OSA. These negatively charged groups enhance the surface charge density of the particles, expanding the thickness of the electrical double layer surrounding the particles and increasing the electrostatic repulsion between particles [25]. According to the electrical double layer theory, a thicker double layer strengthens electrostatic repulsion between particles, effectively counteracting van der Waals attractive forces to prevent irreversible aggregation. This ensures uniform dispersion of particles in aqueous solutions—a critical prerequisite for their application as Pickering emulsion stabilizers. Ferreira et al. [26] also observed a similar trend when modifying sugarcane bagasse-derived CNC with adipic acid: the zeta potential of modified CNC increased significantly due to the introduction of carboxyl groups, improving the dispersion stability of the particles. This consistency confirms the reliability of the zeta potential results in this study and supports the conclusion that OSA modification enhances the dispersion stability of bamboo shoot-derived CNC by regulating surface charge density and electrical double layer thickness.

### 3.6. Contact Angle Analysis

**Figure 5:**
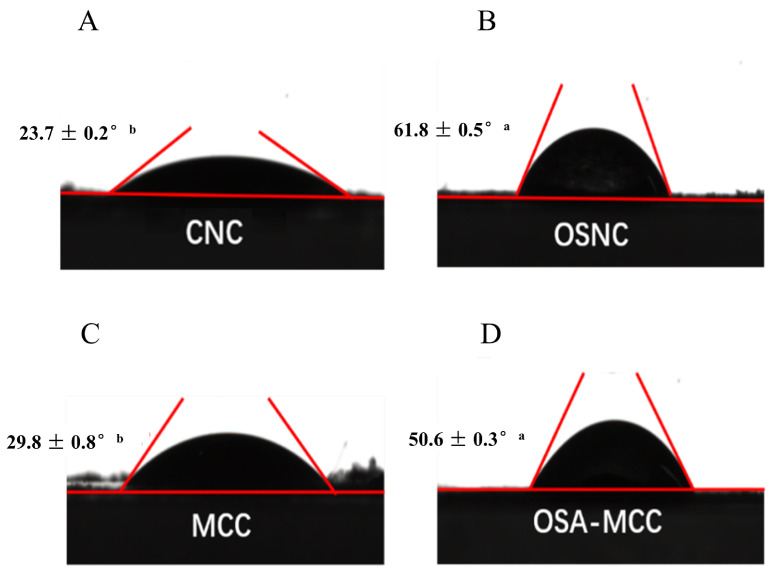
Contact angles of water droplets on the surface of films of bamboo shoot-derived CNC (**A**), OSA-modified CNC (OSNC, (**B**)), MCC (**C**), and OSA-modified MCC (OSA-MCC, (**D**)). Different lowercase letters (a, b) indicate significant differences (*p* < 0.05) between unmodified and OSA-modified samples.

The contact angle directly reflects the wettability of solid particles, and since wettability determines both the particles’ ability to adsorb at the oil-water interface and their performance in stabilizing Pickering emulsions, measuring the contact angle is a key step in verifying the particles’ potential as Pickering emulsion stabilizers. Figure 5 depicts the distinct changes in contact angles exhibited by water droplets when in contact with the surfaces of unmodified versus modified cellulose films, providing direct visual evidence of wettability alterations induced by the modification process. The contact angle of unmodified bamboo shoot CNC is 23.7 ± 0.2°, and that of MCC is 29.8 ± 0.8°, indicating that both unmodified cellulose particles have strong hydrophilicity. This strong hydrophilicity is due to the large number of hydroxyl groups on the cellulose surface, which form hydrogen bonds with water molecules, making it difficult for the particles to migrate to and adsorb at the oil-water interface [27].

After OSA modification, the contact angle of OSA-MCC increases to 50.6 ± 0.3°, and that of OSNC increases to 61.8 ± 0.5° (*p* < 0.05), representing a significant improvement in hydrophobicity. According to Young’s equation, a contact angle in the range of 30° to 150° signifies that particles possess balanced amphiphilic properties, allowing them to adsorb irreversibly at the oil-water interface [28]. Notably, the contact angle of OSNC (61.8°) is lower than that of OSA-modified sweet potato residue cellulose (86.51° to 92.41°) [7] and OSA-modified cotton CNC (82.1° to 85.0°) [11], indicating OSNC has moderate amphiphilicity compared to these OSA-modified celluloses. This discrepancy could be due to the differences in measurement setups of contact angle. Even so, OSNC’s 61.8° still confirms balanced amphiphilicity, sufficient for Pickering emulsion stabilization. This enhanced balance enables OSNC to adsorb more effectively at the oil-water interface, thereby facilitating the formation of a stable interfacial film. The improvement in hydrophobicity after modification is due to the introduction of OSA long-chain alkenyl groups. These hydrophobic chains reduce the surface energy of the particles, weakening the interaction between particles and water molecules, while the retained hydrophilic carboxylate groups ensure that the particles can still be dispersed in the aqueous phase. This “hydrophilic-hydrophobic balance” is the core reason for the improved emulsifying performance of OSNC.

### 3.7. Thermogravimetric Analysis (TGA)

**Figure 6:**
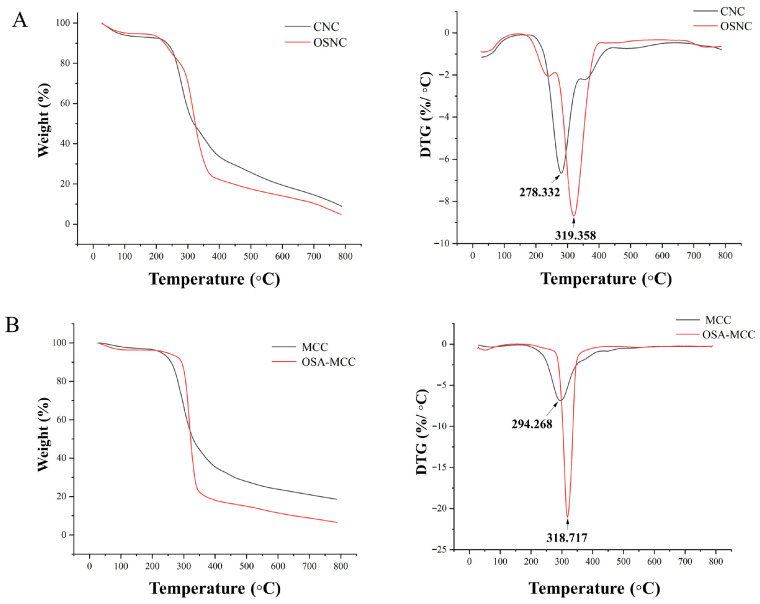
Thermogravimetric (TGA) and derivative thermogravimetric (DTG) curves of bamboo shoot-derived CNC and OSA-modified CNC (OSNC, (**A**)), as well as MCC, and OSA-modified MCC (OSA-MCC, (**B**)).

Thermal stability is a critical indicator for evaluating the applicability of modified cellulose particles in food processing (e.g., pasteurization, baking), where moderate heating is often required. Figure 6 shows the thermogravimetric (TGA) and derivative thermogravimetric (DTG) curves of unmodified and modified cellulose samples. For unmodified bamboo shoot CNC and MCC, the thermal decomposition process can be divided into three stages: (1) Weight loss below 100 °C: This stage is attributed to the evaporation of adsorbed water and crystalline water. The weight loss of bamboo shoot CNC in this stage is approximately 8%, while that of MCC is approximately 5%, which is due to the larger specific surface area of bamboo shoot CNC and its stronger water adsorption capacity. (2) Rapid weight loss stage (200–350 °C): This is the main decomposition stage of cellulose, involving the cleavage of glycosidic bonds, dehydration, and carbonization. The maximum decomposition temperature (Tmax) of bamboo shoot CNC is 278 °C, and that of MCC is 294 °C. (3) Slow weight loss stage above 350 °C: This stage corresponds to the decomposition of residual carbonaceous materials. At 800 °C, the residual weight of bamboo shoot CNC is 8.84%, and that of MCC is 18.53%. After OSA modification, the thermal stability of the particles is significantly improved. The Tmax of OSNC increases to 319.3 °C, and that of OSA-MCC increases to 318.7 °C. This enhancement arises from two key molecular mechanisms: First, ester bonds (-COO^−^) formed between OSA and cellulose have higher bond energy than the hydrogen bonds dominating unmodified cellulose, requiring more thermal energy to cleave during decomposition. Second, the grafted OSA alkyl chains restrict rotation of the cellulose molecular backbone, increasing chain rigidity and reducing conformational entropy—this structural constraint delays the onset of thermal degradation by inhibiting chain relaxation at elevated temperatures [29]. Consistent with these mechanisms, the DTG curves show slower weight loss rates for OSNC and OSA-MCC in the 200–350 °C range, confirming their enhanced resistance to thermal decomposition. At 800 °C, residuals of OSNC (4.68%) and OSA-MCC (6.48%) are lower than unmodified samples, as the OSA-derived aliphatic segments (C8 alkenyl groups) decompose more completely at high temperatures compared to the aromatic carbonaceous residues of unmodified cellulose. The improved thermal stability of OSNC ensures its applicability in food processing; the enhanced ester bond energy and chain rigidity prevent structural collapse, preserving its emulsifying performance and avoiding thermal degradation byproducts that could affect food quality.

## 4. Conclusions

In summary, this study validates the feasibility of preparing OSNC from bamboo shoot bases via acid hydrolysis and OSA modification. The resulting OSNC, with its balanced amphiphilicity, excellent dispersion stability, and improved thermal stability, lays a solid foundation for subsequent research on its application in stabilizing food-grade Pickering emulsions and delivering lipophilic functional factors (e.g., curcumin). Furthermore, this work promotes the high-value utilization of bamboo shoot processing by-products, aligning with the goals of circular agriculture and green material development in the food industry.

## Figures and Tables

**Table 1:** Degree of Substitution of OSNC and OSA-MCC.

Sample	Cellulose:OSA	DS
OSNC	1:0.175	0.019 ± 0.02 ^c^
	1:0.200	0.026 ± 0.01 ^b^
	1:0.225	0.029 ± 0.01 ^a^
	1:0.250	0.030 ± 0.02 ^a^
OSA-MCC	1:0.225	0.024 ± 0.02 ^b^

Different lowercase letters (^a^, ^b^, ^c^) indicate significant differences among different samples (*p* < 0.05).

## Data Availability

The original contributions presented in this study are included in the article. Further inquiries can be directed to the corresponding authors.

## Abbreviations

CNC	Cellulose Nanocrystals
OSA	Octenyl Succinic Anhydride
OSNC	OSA-Modified Bamboo Shoot CNC
MCC	Microcrystalline Cellulose
DS	Degree of Substitution
AGU	Anhydroglucose Unit
FTIR	Fourier Transform Infrared Spectroscopy
XRD	X-Ray Diffraction
SEM	Scanning Electron Microscopy
TGA	Thermogravimetric Analysis
DTG	derivative thermogravimetric
